# Synergistic Regulation of Nitrogen-Doped Carbon Coating and Pseudocapacitive Kinetics in TiO_2_ Nanofibers for Enhanced Sodium-Ion Storage

**DOI:** 10.3390/molecules31091418

**Published:** 2026-04-24

**Authors:** Fei Guo, Liang Xie, Liangquan Wei, Jinmei Du, Shaohui Zhang, Yuanmiao Xie, Baosheng Liu

**Affiliations:** Liuzhou Key Laboratory for New Energy Vehicle Power Lithium Battery, School of Electronic Engineering, Guangxi University of Science and Technology, Liuzhou 545006, China

**Keywords:** sodium-ion batteries, TiO_2_ nanofibers, nitrogen-doped carbon, pseudocapacitance, electrospinning

## Abstract

Sodium-ion batteries (SIBs) represent a compelling alternative to lithium-ion batteries for grid-scale energy storage, owing to the high natural abundance and low cost of sodium resources, as well as their strategic alignment with national energy security priorities. Nevertheless, the sluggish Na^+^ diffusion kinetics and limited specific capacity of anode materials continue to impede practical deployment. Herein, nitrogen-doped carbon-coated TiO_2_ nanofibers (TiO_2_/C-N) were rationally engineered through a facile electrospinning route integrated with synergistic defect and coating engineering. The in situ-formed N-doped carbon shell establishes a continuous, high-conductivity electron-transport network while simultaneously buffering volumetric strain during repeated (de)sodiation, thereby preserving long-term structural integrity. Electrochemical assessments demonstrate that the TiO_2_/C-N electrode delivers a reversible specific capacity of 233.64 mAh g^−1^ at 0.1 A g^−1^ (initial Coulombic efficiency 54.13%). Quantitative kinetic analysis reveals a pronounced pseudocapacitive contribution of 41.4% at 1.2 mV s^−1^, confirming a surface-controlled Na^+^ storage pathway that markedly enhances rate capability. Moreover, the electrode retains 245.5 mAh g^−1^ after 150 cycles at 1 A g^−1^, underscoring exceptional cycling stability. This work elucidates the synergistic regulation of N-doped carbon coating and pseudocapacitive kinetics in TiO_2_-based anodes, offering a robust design strategy for high-rate, long-cycle-life SIB anodes.

## 1. Introduction

Sodium-ion batteries (SIBs) have attracted considerable attention as promising alternatives to lithium-ion batteries because of their abundant sodium resources, low cost, and environmental friendliness [1,2]. However, their practical application is hindered by several limitations of anode materials, such as low specific capacity, slow sodium-ion migration kinetics, poor electronic conductivity, and insufficient cycling stability.

Among various anode materials, titanium dioxide (TiO_2_) has been widely investigated due to its unique advantages. First, TiO_2_ is abundant and environmentally benign. Second, compared with the rutile phase, anatase TiO_2_ possesses a relatively more open crystal structure and exhibits a small volume change (<4%) during the lithiation/delithiation process, which effectively alleviates mechanical stress. It should be noted that among various TiO_2_ polymorphs, TiO_2_(B) is generally considered to possess a more open framework that is highly favorable for ion insertion and diffusion, and it has demonstrated superior performance particularly in lithium-ion batteries [3,4]. However, due to its relatively complex synthesis and structural instability, anatase TiO_2_ remains more widely studied, especially when combined with surface modification strategies. Third, the relatively suitable sodium insertion potential (~0.7 V vs. Na/Na^+^) can suppress sodium dendrite formation and improve battery safety [5]. Nevertheless, the practical performance of TiO_2_ is still limited by its intrinsically low electronic conductivity (~10^−12^ S cm^−1^) and relatively low practical capacity (typically below 200 mAh g^−1^ compared with the theoretical capacity of ~335 mAh g^−1^) [6].

To improve the sodium storage performance of TiO_2_, several strategies have been explored. One effective approach is nanostructure engineering. Nanostructured materials can shorten ion diffusion paths; however, complicated synthesis processes often limit large-scale applications. Among various nanostructures, TiO_2_ nanofibers prepared by electrospinning are attractive due to their simple and scalable fabrication process [7,8,9]. Liu et al. demonstrated that TiO_2_ nanowire structures can significantly enhance the sodium storage capacity in SIB systems [10].

Another widely used strategy is conductive coating design [11,12], which can construct conductive networks and suppress particle aggregation. However, conventional carbon coatings often fail to simultaneously optimize electronic conductivity and surface-active sites. In addition, defect engineering, involving oxygen vacancies [13,14,15,16,17] and titanium vacancies, for example [18,19,20], has been explored to enhance electronic conductivity and provide additional sodium storage sites. Nevertheless, single defects may gradually annihilate during long-term cycling, leading to capacity degradation.

Heteroatom doping, including nitrogen [21], carbon [22], sulfur [23,24], phosphorus [25,26,27], and boron [28,29], has also been widely studied. Doping can effectively tune the electronic structure and improve interfacial charge transfer. However, single-element doping usually provides limited improvement in ion diffusion kinetics.

In this work, nitrogen-doped carbon-coated TiO_2_ nanofibers were rationally designed and synthesized via a facile electrospinning strategy combined with controlled thermal treatment. The introduction of a nitrogen-doped carbon shell is expected to enhance electronic conductivity, provide additional electroactive sites, and facilitate Na^+^ transport. Comprehensive structural and chemical characterizations, including X-ray diffraction (XRD), electron paramagnetic resonance (EPR), and X-ray photoelectron spectroscopy (XPS), were employed to elucidate the defect structure and chemical states of the materials. Meanwhile, systematic electrochemical measurements, together with kinetic analysis based on cyclic voltammetry and electrochemical impedance spectroscopy, were conducted to clarify the sodium storage behavior and the pseudocapacitive contribution. This study aims to provide deeper insight into the synergistic regulation of defect engineering and heteroatom-doped carbon coating in TiO_2_-based anodes, offering a rational strategy for designing high-performance electrode materials for sodium-ion batteries.

## 2. Results and Discussion

**Figure 1:**
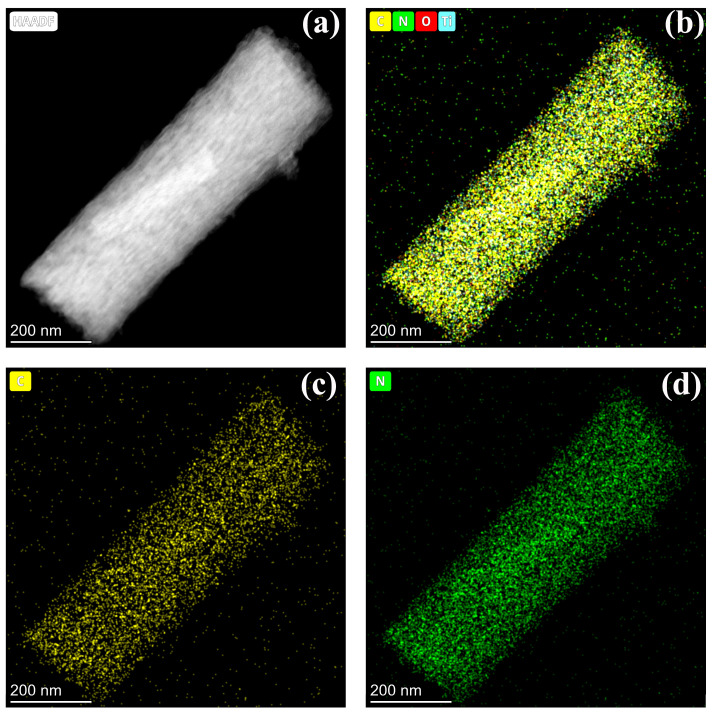
Morphological and elemental characterization of TiO_2_/C-N. (**a**) HAADF-STEM image of a single nanorod. (**b**–**d**) EDS mapping demonstrating the uniform distribution of Carbon (yellow), Nitrogen (green), Oxygen, and Titanium throughout the structure.

The morphology and microstructure of the as-prepared samples were initially investigated using transmission electron microscopy (TEM) and high-resolution TEM (HRTEM). As illustrated in Figure 1, the TiO_2_/C-N sample exhibits a uniform one-dimensional nanofiber morphology resulting from the electrospinning process. These nanofibers possess diameters ranging from approximately 80 to 120 nm, with lengths extending to several tens of micrometers. The smooth surfaces and continuous fibrous structures indicate that the electrospinning process successfully yielded well-dispersed nanofibers without significant agglomeration. A thin carbon layer can be clearly observed surrounding the TiO_2_ nanocrystals. Based on measurements from multiple HRTEM regions, the thickness of the carbon coating is estimated to be approximately 2–5 nm. This nanoscale coating is expected to enhance electronic conductivity while preserving efficient ion transport.

**Figure 2:**
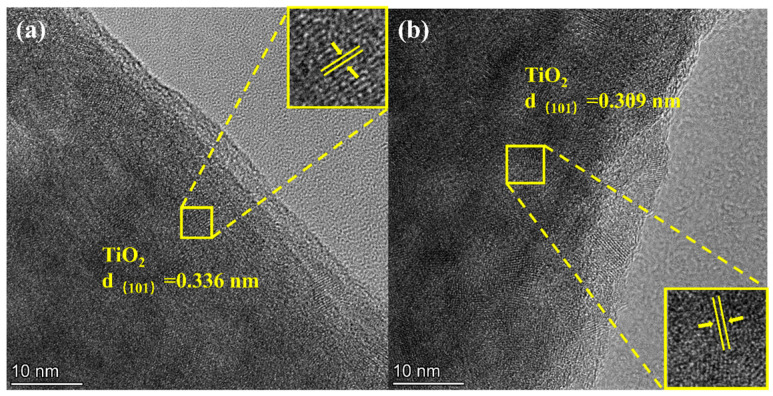
HRTEM images showing TiO_2_ lattice fringes of (**a**) TiO_2_/C-N and (**b**) TiO_2_/C.

Notably, the successful incorporation of nitrogen is believed to induce localized lattice distortion within the TiO_2_ structure. As shown in Figure 2, the measured lattice spacings of 0.309 nm and 0.336 nm for TiO_2_/C-N are slightly smaller than the standard value of 0.352 nm for the anatase TiO_2_ (101) plane. This contraction can be rationally attributed to the synergistic effects of nanoscale confinement, point defects, and strong interfacial interaction between the TiO_2_ nanocrystals and the surrounding N-doped carbon shell. Such structural modulation is commonly observed in heteroatom-doped carbon-coated oxides and is expected to facilitate Na^+^ diffusion while enhancing mechanical robustness during cycling.

Elemental distribution was further examined using energy-dispersive X-ray spectroscopy (EDS) mapping. The elemental mapping results reveal that C and N elements are homogeneously distributed throughout the nanofiber matrix. It should be noted that EDS analysis is semi-quantitative in nature; therefore, the precise elemental composition is further analyzed by XPS, as discussed below. Such a uniform distribution suggests that the carbon and nitrogen species originating from the organic precursor were carbonized in situ during the thermal treatment, forming a stable TiO_2_/C-N heterostructure, which can create additional active sites for sodium storage and facilitate ion transport during electrochemical reactions.

**Figure 3:**
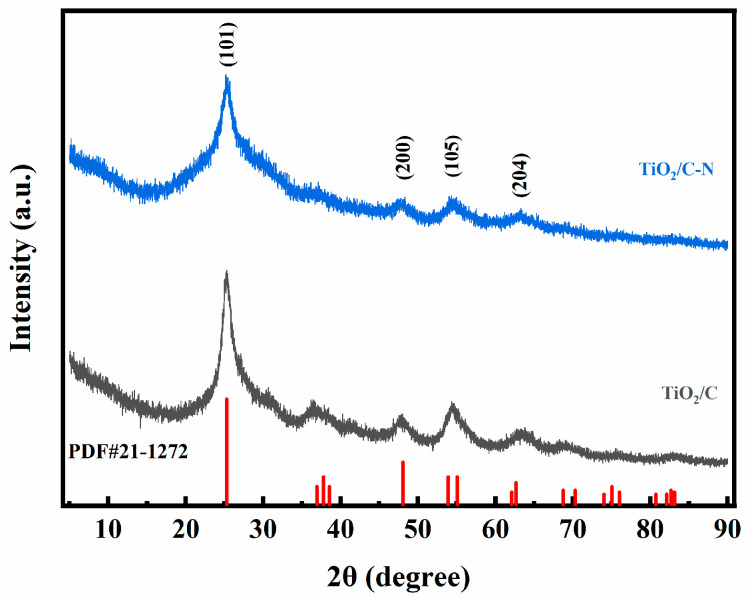
XRD patterns of TiO_2_/C-N and TiO_2_/C samples. The diffraction peaks are indexed to the anatase phase of TiO_2_ (PDF#21-1272).

The crystal structures of the samples were analyzed using X-ray diffraction (XRD), and the results are presented in Figure 3. The diffraction peaks of both TiO_2_/C-N and TiO_2_/C samples match well with the standard anatase TiO_2_ phase (PDF#21-1272). A prominent diffraction peak located at approximately 2θ = 25.3° corresponds to the (101) plane of anatase TiO_2_, which is the most characteristic peak of this phase [30]. Additional diffraction peaks appearing at around 38.0°, 48.0°, and 55.0° can be indexed to the (004), (200), and (211) planes of anatase TiO_2_, respectively. No obvious diffraction peaks corresponding to impurity phases such as rutile TiO_2_ are observed, indicating that the synthesized samples possess high phase purity. The relatively sharp diffraction peaks confirm the formation of crystalline anatase TiO_2_ within the nanofiber matrix, with no detectable rutile or impurity phases. This high phase purity ensures reproducible electrochemical behavior in subsequent tests.

**Figure 4:**
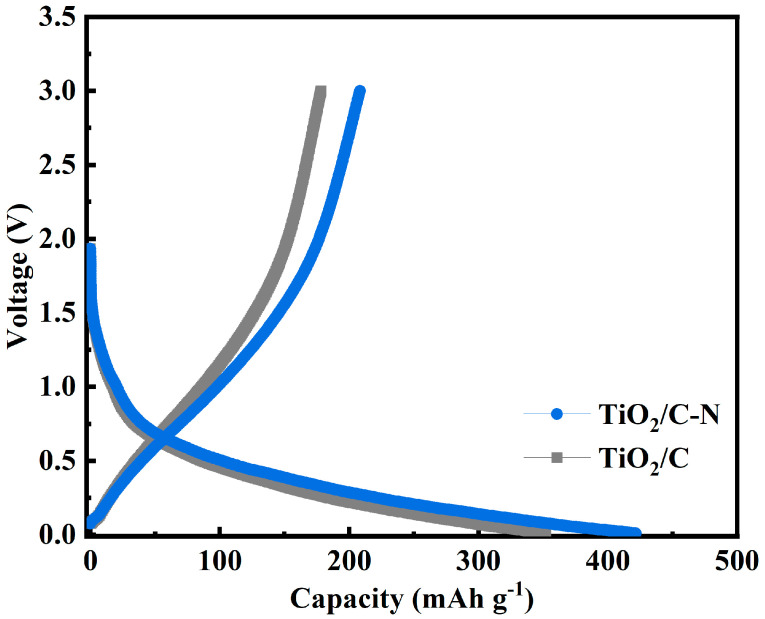
Initial galvanostatic charge–discharge curves of TiO_2_/C-N and TiO_2_/C electrodes at a specific current density.

To evaluate the electrochemical performance of the materials, sodium-ion half cells were assembled and tested. Figure 4 shows the initial galvanostatic charge–discharge profiles of the TiO_2_/C-N and TiO_2_/C electrodes at a current density of 0.1 A g^−1^. The TiO_2_/C-N electrode delivers an initial discharge capacity of 431.63 mAh g^−1^ and a charge capacity of 233.64 mAh g^−1^, corresponding to an initial Coulombic efficiency of 54.13%. In comparison, the TiO_2_/C electrode exhibits an initial discharge capacity of 352.14 mAh g^−1^, a charge capacity of 175.27 mAh g^−1^, and an initial Coulombic efficiency of 49.77%. Furthermore, the presence of a conductive carbon layer can help alleviate irreversible side reactions during the formation of the solid electrolyte interphase (SEI), thereby improving electrochemical reversibility [31]. The enhanced performance of the TiO_2_/C-N electrode compared to TiO_2_/C may be related to differences in the carbon structure and surface chemistry. The enhanced initial Coulombic efficiency and reversible capacity of the TiO_2_/C-N electrode relative to TiO_2_/C can be primarily ascribed to the N-doped carbon layer, which minimizes irreversible SEI formation and improves electronic wiring at the electrode–electrolyte interface. These improvements are further corroborated by the surface chemical analysis presented below.

**Figure 5:**
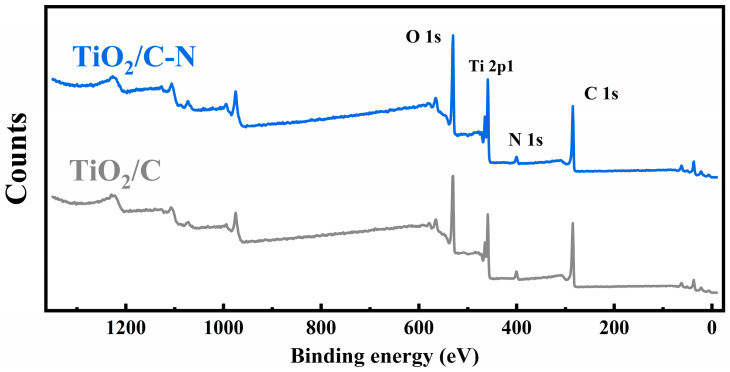
XPS survey spectra of TiO_2_/C-N and TiO_2_/C samples, indicating the presence of Ti, O, C, and N elements.

The surface chemical states of the samples were further analyzed using X-ray photoelectron spectroscopy (XPS). As shown in Figure 5, the survey spectra confirm the presence of Ti, O, C, and N elements in the TiO_2_/C-N sample. The high-resolution spectra provide more detailed information about the chemical states of these elements. The Ti 2p XPS spectra of both samples exhibit characteristic peaks corresponding to Ti^4+^ species, which are consistent with the typical oxidation state of Ti in TiO_2_ [32,33].

**Figure 6:**
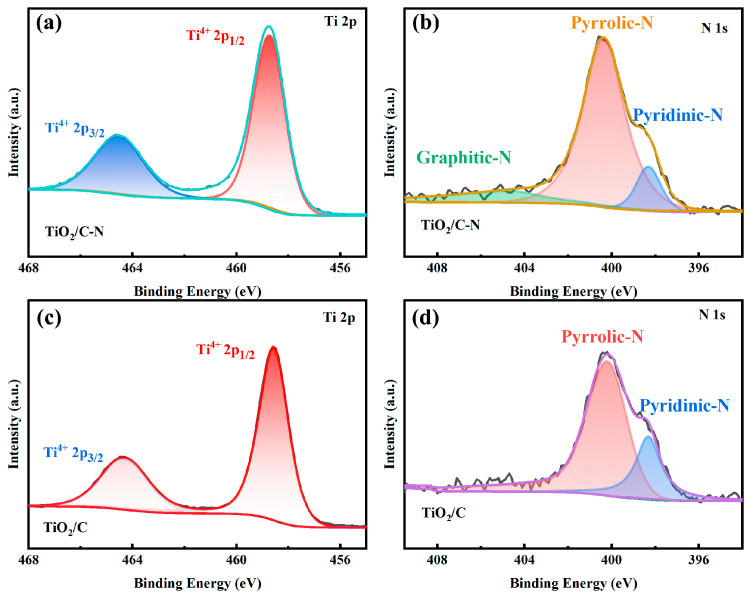
High-resolution XPS spectra of (**a**,**c**) Ti 2p and (**b**,**d**) N 1s for TiO_2_/C-N and TiO_2_/C. The different colored shaded areas represent the deconvoluted peaks corresponding to specific chemical states: Ti^4+^ 2p_1_/_2_ and Ti^4+^ 2p_3_/_2_ in the Ti 2p spectra (**a**,**c**); pyridinic N, pyrrolic N, and graphitic N in the N 1s spectra (**b**,**d**).

The high-resolution N 1s spectra (Figure 6b,d) confirm the presence of nitrogen species in the TiO_2_/C-N sample. Compared with TiO_2_/C, the overall nitrogen signal intensity is slightly higher. In contrast, TiO_2_/C-N showed an increased nitrogen content of 4.3% and a decreased oxygen content of 27.6%. Due to the relatively low signal intensity, a detailed deconvolution of nitrogen species is not performed. Nitrogen doping in carbon materials is known to improve the electrical conductivity and facilitate electron transport within the electrode. Moreover, the nitrogen-doped carbon coating can modify the local electronic structure and enhance the interaction between sodium ions and the electrode surface, thereby improving sodium storage performance. A weak N signal is also observed in the TiO_2_/C sample, which may originate from residual nitrogen-containing species during synthesis or minor environmental contamination. The higher nitrogen content in the TiO_2_/C-N sample confirms the successful introduction of nitrogen into the carbon coating [34].

**Figure 7:**
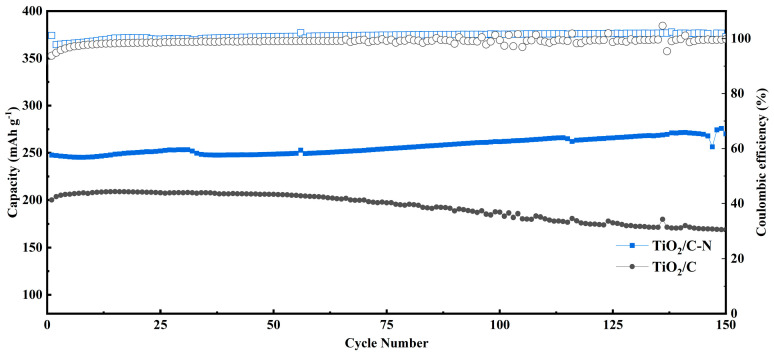
Cycling performance and corresponding Coulombic efficiency of TiO_2_/C-N and TiO_2_/C electrodes over 150 cycles at a current density of 1 A g^−1^. The solid symbols denote the discharge capacities (left axis), while the hollow squares and circles represent the Coulombic efficiencies (right axis) of TiO_2_/C-N and TiO_2_/C, respectively.

The long-term cycling stability of the electrodes was evaluated at a high current density of 1 A g^−1^. After 150 cycles, the TiO_2_/C-N electrode maintains a specific capacity of 245.5 mAh g^−1^, as shown in Figure 7, demonstrating superior durability compared with recently reported TiO_2_-based anodes. For instance, nanoporous anatase TiO_2_ delivers only ~145 mAh g^−1^ at 0.35 A g^−1^, and this drops to ~75 mAh g^−1^ at ~1.8 A g^−1^ [35]. Similarly, conventional carbon-coated TiO_2_ microspheres exhibit comparable capacities near 149 mAh g^−1^ exclusively at low rates around 0.2 A g^−1^ but suffer from severe decay to below 83 mAh g^−1^ at higher currents near 2 A g^−1^ [36]. In stark contrast, our TiO_2_/C-N composite sustains a high capacity of 245.5 mAh g^−1^ even at 1 A g^−1^, indicating that the nitrogen-containing carbon framework significantly enhances both rate capability and structural integrity beyond what is achievable with simple nanostructuring or undoped carbon coating.

**Figure 8:**
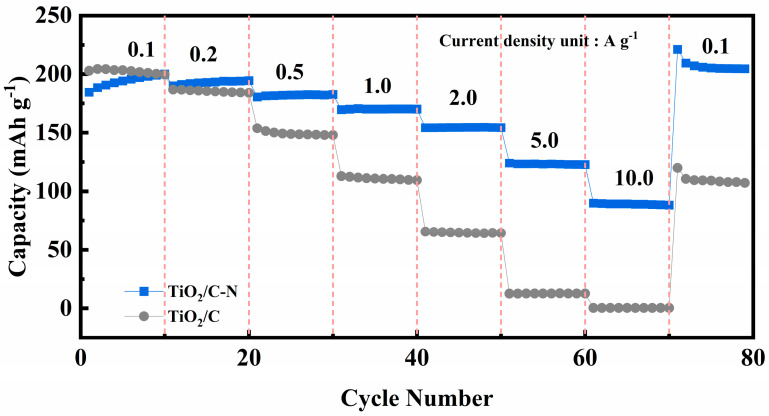
Rate capability of TiO_2_/C-N and TiO_2_/C electrodes at various current densities ranging from 0.1 to 10 A g^−1^.

The rate capability of the electrodes was further investigated to evaluate their high-rate sodium storage performance. As illustrated in Figure 8, the TiO_2_/C-N electrode delivers reversible capacities of 215.55, 205.67, 188.22, 161.21, 121.60, 61.69, and 15.28 mAh g^−1^ at current densities of 0.1, 0.2, 0.5, 1, 2, 5, and 10 A g^−1^, respectively. When the current density returns to 0.1 A g^−1^, the capacity recovers to 227.42 mAh g^−1^, indicating excellent structural reversibility and stability of the electrode. The discrepancy between Figure 7 and Figure 8 originates from differences in testing protocols. The cycling performance shown in Figure 7 reflects stabilized capacity after gradual electrode activation during prolonged cycling at a constant current density. In contrast, the rate capability test in Figure 8 involves stepwise changes in current density over relatively short time intervals, where sufficient activation and stabilization are not achieved at each current level. As a result, the capacity measured at higher current densities is relatively lower than that obtained under steady-state cycling conditions. All electrochemical measurements were performed using freshly assembled cells to ensure the reliability and reproducibility of the results. The improved rate capability can be attributed to the synergistic effect of nitrogen-containing carbon layer, which together enhance electronic conductivity and accelerate sodium-ion diffusion kinetics.

**Figure 9:**
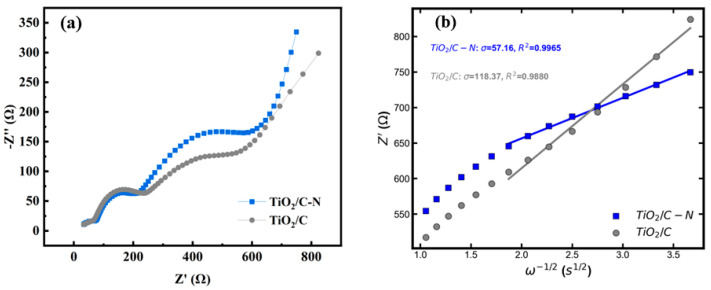
Electrochemical impedance spectroscopy (EIS) analysis after 100 cycles at 1 A g^−1^: (**a**) Nyquist plots and (**b**) the relationship between Z′ and ω^−1/2^ in the low-frequency region for TiO_2_/C-N and TiO_2_/C electrodes.

To further understand the charge-transfer kinetics and ion diffusion behavior, electrochemical impedance spectroscopy (EIS) measurements were conducted after 100 cycles at a current density of 1 A g^−1^. The Nyquist plots shown in Figure 9 exhibit a high-frequency intercept corresponding to the solution resistance, followed by two semicircles in the high-to-medium-frequency region and an inclined line in the low-frequency region [37]. The first semicircle is attributed to the resistance of sodium-ion migration through the surface film (SEI), while the second corresponds to the charge-transfer resistance at the electrode–electrolyte interface. The TiO_2_/C-N electrode displays a smaller semicircle compared to TiO_2_/C, indicating reduced charge-transfer resistance and improved interfacial kinetics, which can be attributed to the enhanced electronic conductivity provided by the nitrogen-doped carbon coating. In contrast, the similar high-frequency intercepts suggest comparable solution and SEI resistances for both samples.

**Figure 10:**
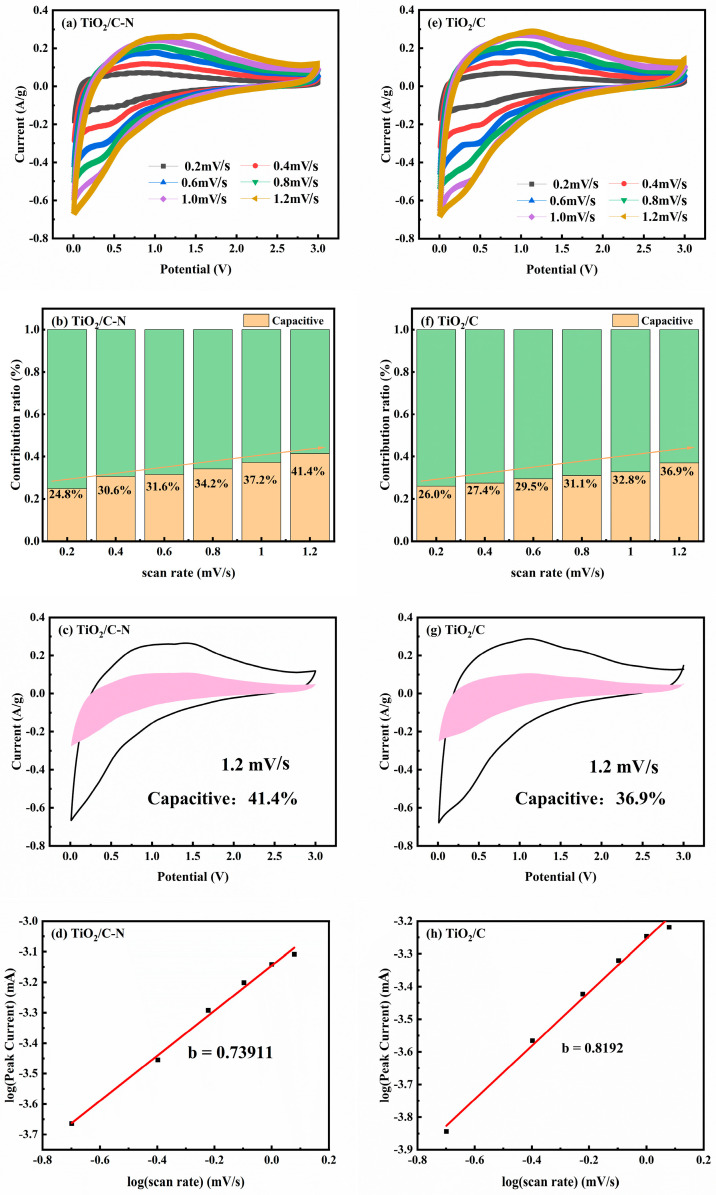
Electrochemical kinetic analysis of TiO_2_/C–N (**a**–**d**) and TiO_2_/C (**e**–**h**): (**a**,**e**) CV curves at different scan rates (0.2–1.2 mV s^−1^); (**b**,**f**) pseudocapacitive contribution ratios; (**c**,**g**) separation of capacitive and diffusion-controlled currents; (**d**,**h**) linear fitting of log(peak current) versus log(scan rate).

In the low-frequency region, Z′ shows a linear dependence on ω^−1^/^2^, characteristic of diffusion-controlled behavior associated with Warburg impedance. Linear fitting was performed to obtain the Warburg coefficient (σ) from the slope of the fitted lines. Based on this, the sodium-ion diffusion coefficient (D_Na_^+^) was calculated according to the following equation: D = R^2^T^2^/(2A^2^n^4^F^4^C^2^σ^2^), where R is the gas constant, T is the absolute temperature, A is the electrode area, n is the number of electrons transferred, F is Faraday’s constant, C is the molar concentration of sodium ions, and σ is the Warburg coefficient [38]. As summarized in Table 1, the TiO_2_/ C-N electrode exhibits a lower σ value and a correspondingly higher D_Na_^+^ compared to TiO_2_/C after 100 cycles at 1 A g^−1^, indicating enhanced sodium-ion diffusion kinetics. This improvement is attributed to the synergistic effect of nitrogen doping and carbon coating, which facilitates electron transport and promotes efficient ion diffusion within the electrode. To further elucidate the sodium storage mechanism, cyclic voltammetry (CV) measurements were carried out at various scan rates, and the results are presented in Figure 10. The CV curves of TiO_2_/C-N and TiO_2_/C recorded at scan rates ranging from 0.2 to 1.2 mV s^−1^ exhibit well-defined redox peaks with only slight shifts in peak position, indicating low polarization and favorable electrochemical kinetics.

The charge storage mechanism was quantitatively analyzed using the power-law relationship i = aν^b^ [41]. The b-values for the anodic peak were determined to be 0.739 for TiO_2_/C-N and 0.819 for TiO_2_/C (Figure 10d,h), indicating that both electrodes operate via a mixed diffusion- and surface-controlled process. Although the b-value of TiO_2_/C-N is slightly lower, its integrated pseudocapacitive contribution—calculated via i = k_1_v + k_2_v^1/2^ [42]—reaches 41.4% at 1.2 mV s^−1^, significantly higher than the 36.9% observed for TiO_2_/C (Figure 10b,f). This apparent discrepancy is well-documented in the literature: b-values primarily reflect peak-current behavior, whereas the capacitive contribution quantifies the total charge stored via surface processes. The enhanced capacitive fraction in TiO_2_/C-N originates from the additional redox-active sites provided by pyridinic-N species and improved interfacial conductivity, which synergistically accelerate surface-controlled Na^+^ storage. These kinetic insights are fully consistent with the reduced charge-transfer resistance and elevated D_Na_^+^ values obtained from EIS. Overall, the markedly enhanced electrochemical performance of TiO_2_/C-N can be ascribed to the synergistic integration of the N-doped carbon coating and the one-dimensional nanofiber architecture. The conductive carbon network provides rapid electron pathways, while the continuous fibrous morphology offers short Na^+^ diffusion lengths and mechanical buffering, collectively enabling fast pseudocapacitive sodium storage and long-term structural stability.

## 3. Materials and Methods

TiO_2_ nanofibers were synthesized via an electrospinning method. In a typical procedure, 5 mL of tetrabutyl titanate (TBOT) and 1.2 g of polyacrylonitrile (PAN) were dissolved in a mixed solvent consisting of 20 mL ethanol and acetic acid (volume ratio 7:3). The solution was magnetically stirred at 40 °C for 3 h at 400 rpm to obtain a homogeneous precursor solution. Subsequently, 5 mL of diisopropyl azodicarboxylate (DIPA) was introduced under vigorous stirring and the mixture was further stirred for 10 min to obtain the electrospinning precursor.

The precursor solution was transferred into a syringe equipped with a 21-gauge needle. Electrospinning was carried out at an applied voltage of 20 kV with a feeding rate of 0.0020 mm∙s^−1^ and a collector distance of 11 cm. The obtained electrospun membrane was dried at 60 °C for 8 h and subsequently calcined at 500 °C for 12 h under an argon atmosphere with a heating rate of 5 °C min^−1^ to obtain carbon-coated TiO_2_ nanofibers (TiO_2_/C-N). For comparison, TiO_2_/C samples were prepared following the same procedure without the addition of DIPA during precursor preparation.

The crystalline structure of the samples was characterized using X-ray diffraction (XRD, Smartlab SE, Rigaku Corporation is headquartered in Akishima, Tokyo, Japan). The morphology and microstructure were investigated by transmission electron microscopy (TEM, FEI Talos F200S, FEI Company (now part of Thermo Fisher Scientific) has its primary electron microscopy manufacturing and R&D facility in Hillsboro, OR, USA). The surface chemical composition and electronic states were analyzed using X-ray photoelectron spectroscopy (XPS, Thermo Fisher ESCALAB 250Xi, Thermo Fisher Scientific Inc. is headquartered in Waltham, MA, USA).

For electrochemical measurements, the working electrode was prepared by mixing 70 wt% active material, 20 wt% Super P conductive carbon, and 10 wt% polyvinylidene fluoride (PVDF) binder in N-methyl-2-pyrrolidone (NMP) to form a homogeneous slurry. Herein, the carbon coating on the TiO_2_ nanofibers mainly enhances local electronic conductivity and structural stability, while Super P serves as a conductive additive to establish an effective electron transport network within the electrode. The slurry was uniformly coated onto copper foil and dried in a vacuum oven at 80 °C for 8 h. The dried electrodes were punched into circular disks with a diameter of 12 mm. The typical mass loading of the active material was approximately 1.25 mg cm^−2^.

CR-2025 coin cells were assembled in an argon-filled glovebox using sodium metal as the counter/reference electrode and Whatman glass fiber as the separator. The electrolyte consisted of 1.0 M NaPF_6_ dissolved in a mixed solvent of ethylene carbonate (EC), dimethyl carbonate (DMC), and ethyl methyl carbonate (EMC) with a volume ratio of 1:2:2. Galvanostatic charge–discharge tests were conducted on a Neware battery testing system within a voltage window of 0.01–3.0 V (vs. Na/Na^+^). Cyclic voltammetry (CV) and electrochemical impedance spectroscopy (EIS) measurements were performed using a CHI760E electrochemical workstation (CH Instruments, Inc., Bee Cave, TX, USA). The data were analyzed using ZView software (version 3.5; Scribner Associates, Inc., Southern Pines, NC, USA).

## 4. Conclusions

In summary, N-doped carbon-coated TiO_2_ nanofibers (TiO_2_/C-N) were successfully fabricated via a scalable electrospinning strategy coupled with in situ heteroatom doping and carbon coating. The resulting core–shell architecture constructs a highly conductive electron-transport network and effectively mitigates mechanical stress during Na^+^ (de)intercalation. Electrochemical characterization demonstrates that the TiO_2_/C-N electrode achieves a reversible specific capacity of 233.64 mAh g^−1^ at 0.1 A g^−1^ (initial Coulombic efficiency 54.13%) and retains 245.5 mAh g^−1^ after 150 cycles at 1 A g^−1^. In addition, a capacity of 161.21 mAh g^−1^ is retained at 1 A g^−1^ in rate-capability testing, with excellent capacity recovery upon returning to low rates. Kinetic analyses confirm a mixed diffusion- and surface-controlled storage mechanism, with the pseudocapacitive contribution reaching 41.4% at 1.2 mV s^−1^—substantially higher than that of the undoped counterpart (36.9%). These enhancements originate from the synergistic effects of the N-doped carbon shell, which simultaneously boosts electronic conductivity, introduces additional active sites, and accelerates Na^+^ diffusion (D_Na_^+^ = 8.47 × 10^−16^ cm^2^ s^−1^). Compared with pristine carbon-coated TiO_2_, the TiO_2_/C-N composite exhibits markedly superior rate capability and cycling endurance. This study provides a clear demonstration of how heteroatom-doped carbon coating and pseudocapacitive engineering can be synergistically harnessed to overcome the intrinsic limitations of TiO_2_ anodes, paving the way for the rational design of high-performance, low-cost SIB electrode materials.

## Figures and Tables

**Table 1:** The sodium ion diffusion coefficient after 100 cycles of 1 Ag^−1^ current.

	TiO_2_/C-N	TiO_2_/C	TiO_2_(R) [39]	N,Se-TiO_2_-1/3 [40]
$D_{{Na}^{+}}$ (cm^2^ S^−1^)	8.47 × 10^−16^	1.98 × 10^−16^	10^−22^	4.0 × 10^−12^

## Data Availability

The data presented in this study are available on request from the corresponding author. The raw/processed data required to reproduce these findings cannot be shared at this time as the data also form part of an ongoing study.
